# The Amplitude-Normalized Area of a Bipolar Electrogram as a Measure of Local Conduction Delay in the Heart

**DOI:** 10.3389/fphys.2020.00465

**Published:** 2020-05-19

**Authors:** Caroline Mendonca Costa, Grace C. Anderson, Veronique M. F. Meijborg, Christopher O’Shea, Michael J. Shattock, Paulus Kirchhof, Ruben Coronel, Steven Niederer, Davor Pavlovic, Tarvinder Dhanjal, James Winter

**Affiliations:** ^1^School of Biomedical Engineering and Imaging Sciences, King’s College London, London, United Kingdom; ^2^School of Cardiovascular Medicine & Sciences, King’s College London, London, United Kingdom; ^3^Department of Experimental Cardiology, Academic Medical Center, Amsterdam, Netherlands; ^4^Department of Cardiology, UHB NHS Foundation Trust, Institute of Cardiovascular Science, University of Birmingham, Birmingham, United Kingdom; ^5^Department of Cardiology, SWBH NHS Trust, Institute of Cardiovascular Science, University of Birmingham, Birmingham, United Kingdom; ^6^LIRYC, Heart Arrhythmia and Modeling Institute, Pessac, France; ^7^Department of Cardiology, University Hospitals Coventry and Warwickshire, Coventry, United Kingdom

**Keywords:** electrophysiology, bipolar electrogram, conduction delay, substrate mapping, cardiac arrhythmia, cardiac mapping

## Abstract

**Background:**

Re-entrant ventricular tachycardia may be non-inducible or haemodynamically compromising, requiring assessment of the electrophysiological properties of the myocardium during sinus rhythm (i.e., substrate mapping). Areas of heart tissue with slow conduction can act as a critical isthmus for re-entrant electrical excitation and are a potential target for ablation therapy.

**Aim:**

To develop and validate a novel metric of local conduction delay in the heart, the amplitude-normalized electrogram area (norm_EA).

**Methods:**

A computational model of a propagating mouse action potential was used to establish the impact of altering sodium channel conductance, intracellular conductivity, fibrosis density, and electrode size/orientation on bipolar electrogram morphology. Findings were then validated in experimental studies in mouse and guinea pig hearts instrumented for the recording of bipolar electrograms from a multipolar linear mapping catheter. norm_EA was calculated by integrating the absolute area of a bipolar electrogram divided by the electrogram amplitude. Electrogram metrics were correlated with the local conduction delay during sodium channel block, gap junction inhibition, and acute ischemia.

**Results:**

In computational simulations, reducing sodium channel conductance and intracellular conductivity resulted in a decrease in signal amplitude and increase in norm_EA (reflecting a broadening of electrogram morphology). For larger electrodes (3 mm diameter/7.1 mm^2^ area), the change in norm_EA was essentially linear with the change in local conduction delay. Experimental studies supported this finding, showing that the magnitude of change in norm_EA induced by flecainide (1–4 μM), carbenoxolone (10–50 μM), and low-flow ischemia (25% of initial flow rate) was linearly correlated with the local conduction delay in each condition (*r*^2^ = 0.92). Qualitatively similar effects were observed in guinea pig hearts perfused with flecainide. Increasing fibrosis density in the computational model also resulted in a decrease in signal amplitude and increase in norm_EA. However, this remains to be validated using experimental/clinical data of chronic infarct.

**Conclusion:**

norm_EA is a quantitative measure of local conduction delay between the electrode pair that generates a bipolar electrogram, which may have utility in electrophysiological substrate mapping of non-inducible or haemodynamically compromising tachyarrhythmia.

## Introduction

Substrate mapping of the ventricular myocardium is an electrophysiological mapping modality that is commonly applied when the culprit arrhythmia cannot be induced or is hemodynamically compromising (Pedersen et al., 2014; Priori et al., 2015). It is typically performed during electrical pacing or in sinus rhythm (Pedersen et al., 2014). Substrate mapping metrics such as bipolar electrogram voltage (amplitude), fractionation, and late/split/double potentials are used to define regions of tissue that are deemed critical to the initiation and maintenance of re-entrant tachycardia (i.e., the isthmus of the circuit) and are therefore a target for radiofrequency ablation therapy (Josephson and Anter, 2015). The conduction of electrical impulses in such regions is typically slow when compared to the normal myocardium, usually as a result of injury and tissue remodeling. For example, in ventricular scar formed after myocardial infarction, conduction is typically delayed by the tortuous pattern of activation through surviving myocardial fibers found within the scar tissue (de Baker et al., 1990).

A means to assess regions of tissue where there is abnormally slow conduction during substrate mapping could allow better targeting of ablation therapy. One already established metric is the duration of the activation components of the bipolar electrogram. Theoretically, an increase in the conduction time (greater conduction delay) between the two recording electrodes results in a broader electrogram morphology (Venkatachalam et al., 2011). Indeed, it is already known that prolonged bipolar electrogram duration is a characteristic of heart disease and of electrograms recorded from and around ventricular scar tissue (Cassidy et al., 1984a, b, 1986; Untereker et al., 1985; Vassallo et al., 1986). However, electrogram duration is not a widely used metric in substrate mapping procedures. This may reflect the ambiguity in defining the start and end of the electrogram complexes, which is particularly relevant for low-amplitude-signals, as well as the fact that automated measurements of electrogram duration are susceptible to errors caused by signal artifacts. To address these limitations, we sought to develop an alternative, algorithmically calculable and quantitative metric of conduction delay with potential application in electrophysiological substrate mapping procedures.

The total area of the activation components of a bipolar electrogram is a function of electrogram amplitude, electrogram duration, the number of peaks and troughs within the signal and the diameter of the recording electrode. Delayed or slowed electrical conduction in the tissues underlying the recording electrodes would be expected to both reduce signal amplitude and prolong electrogram duration, acting to decrease and increase the area of the electrogram, respectively. We hypothesized that the absolute electrogram area (EA) normalized to the signal amplitude, herein referred to as normalized EA (norm_EA), could be used as a quantitative index of local conduction delay. The present study uses a combination of computational modeling and experimental studies in mammalian hearts to test this hypothesis.

## Materials and Methods

### Computational Modeling

**FIGURE 1:**
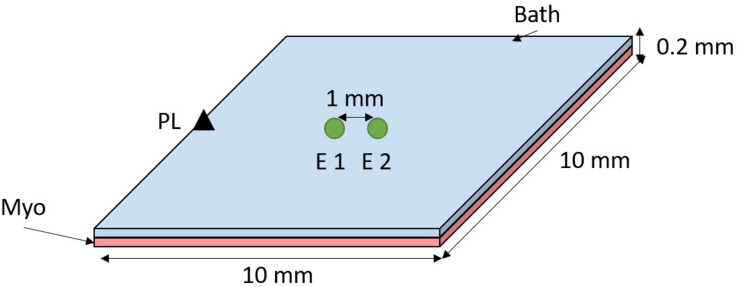
Computational model setup. A sheet of myocardium (pink) is covered by a thin layer of bath (blue). The tissue was paced at the middle left edge (black triangle). Bipolar electrograms were measured at the center of the tissue by subtracting the signals from electrodes (E) 1 and 2–1 mm apart (edge-to-edge).

A 3D mesh of hexahedral elements was created representing a sheet of myocardium covered by a thin layer of bath (see Figure 1). Myocardium and bath dimensions are each 10 × 10 × 0.01 mm. Mesh elements have a mean resolution of 0.01 mm. Cardiac electrophysiology was simulated using the cardiac bidomain model of action potential propagation coupled with the Bondakenko (Bondarenko et al., 2004) model of the action potential of mouse ventricular myocytes. Simulations were run using the Cardiac Arrhythmia Research Package (CARP) (Vigmond et al., 2003). Tissue conductivities were tuned (Costa et al., 2013) to yield a conduction velocity of ∼0.75 m/s, comparable with experimentally measured values in mouse heart (Boukens et al., 2013). Isotropic intracellular and extracellular conductivities of 0.7 and 1.03 S/m, respectively, were assigned to the bidomain model.

Bipolar electrograms were recorded from the center of the tissue with (unless stated otherwise) 0.1 mm electrode diameter and electrode spacing of 1 mm, see Figure 1. Pacing stimuli were delivered to the tissue edge to generate a wavefront that propagated parallel to the orientation of the recording electrodes. The effect of conduction slowing on electrogram morphology was investigated by decreasing the sodium channel conductance (gNa) and the intracellular conductivity (σ_*i*_), separately, in a stepwise manner from 100 to 10% of the initial model parameters.

It is known that electrode diameter affects electrogram morphology due to spatial averaging (Jacquemet and Henriquez, 2009). We have investigated the impact of electrogram diameter in our simulations by averaging the signal of mesh nodes within a circle (as depicted in Figure 1–green circles) with diameter varying from 0.1 to 3.0 mm in steps of 0.1 mm. Spacing between the electrodes was preserved at 1 mm (edge-to-edge).

Fibrosis was included in a circular region (radius = 2 mm) in the middle of the slab. Different fibrosis densities were modeled, namely 20, 40, 60, 80, and 100%. This was achieved by randomly selecting the desired percentage of finite elements within the fibrosis region and removing these from the intracellular grid but not the extracellular grid, thus allowing, extracellular signals (electrograms) to be computed. This effectively models fibrosis as non-conducting tissue, as done previously (Balaban et al., 2018).

Activation times were measured as the time at which the local action potential reached maximum upstroke velocity (dV/dt). Action potentials were averaged according to electrode size, as done for electrograms.

### Experimental Studies

#### Animal Welfare/Ethics

All procedures were undertaken in accordance with ethical guidelines set out by the United Kingdom Animals (Scientific Procedures) Act 1986 and Directive 2010/63/EU of the European Parliament on the protection of animals used for scientific purposes. Studies conformed to the Guide for the Care and Use of Laboratory Animals published by the United States National Institutes of Health under assurance number A5634-01. The procedures had been approved by the University of Birmingham and King’s College London Animal Welfare and Ethical Review Boards.

#### Isolated Heart Studies

Mouse (C57/BL6, 25–30 g, Charles River, United Kingdom) hearts were isolated under isoflurane-induced anesthesia (4 in 100% O_2_) with concomitant intraperitoneal injection of heparin (100 units injected 5-min before heart isolation). Hearts were Langendorff-perfused via the aorta at a perfusion pressure of 70–80 mmHg with an oxygenated (95% O_2_ 5% CO_2_) crystalloid buffer, containing (in mM): NaCl 114, KCl 4, CaCl 1.4, NaHCO_3_ 24, NaH_2_PO_4_ 1.1, glucose 11.0 and sodium pyruvate 1.0 (pH 7.4, 37°C).

Guinea pig hearts (Dunkin Hartley, Marshall BioResources, 450–550 g) were isolated under sodium pentobabitone (160 mg/kg, i.p.) induced anesthesia with concomitant injection of heparin (150 units). Hearts were Langendorff perfused via the aorta at a perfusion pressure of 60–70 mmHg with an oxygenated (ibidem) crystalloid buffer, containing (in mM): NaCl 114, KCl 4, CaCl 1.8, NaHCO_3_ 24, NaH_2_PO_4_ 1.1, glucose 11.0 and sodium pyruvate 1.0 (pH 7.4, 37°C).

#### Protocols

An eight pole electrophysiological mapping catheter was inserted into the left ventricular lumen via a small incision in the left atrium. Between four and five unipolar electrograms (poles 4–8) were recorded from the endocardial surface of the left ventricular free wall. Reference and ground electrodes were placed in the perfusion chamber (∼3 cm from the heart). The catheter diameter was 1 mm and electrode height was 1 mm, giving a total electrode surface area of 3.14 mm^2^. Three to four bipolar electrograms were calculated from adjacent poles (1 mm spacing) according to the method of Blanchard et al. (1988). All data were digitized at 4 kHz with 0.2 Hz high-pass and 1000 Hz low-pass filters (OctalBioamp and PowerLab 16s, ADInstruments, Australia).

Mouse hearts were paced at 3× the diastolic threshold via the two distal poles on the mapping catheter (endocardial pacing, 420 bpm, 1ms pulse duration). Interventions to alter ventricular conduction were (i) 3-min of low-flow global ischemia (25% of initial flow rate), (ii) increasing concentrations of the sodium channel blocker flecainide (1–4 μmol/L), and (iii) increasing concentrations of the gap junction inhibitor carbenoxolone (10–50 μmol/L).

Guinea pig hearts were allowed to beat at their intrinsic (sinus) rate and after a baseline stability period, were perfused with flecainide (4 μmol/L).

### Calculation of Normalized Electrogram Area (norm_EA)

**FIGURE 2:**
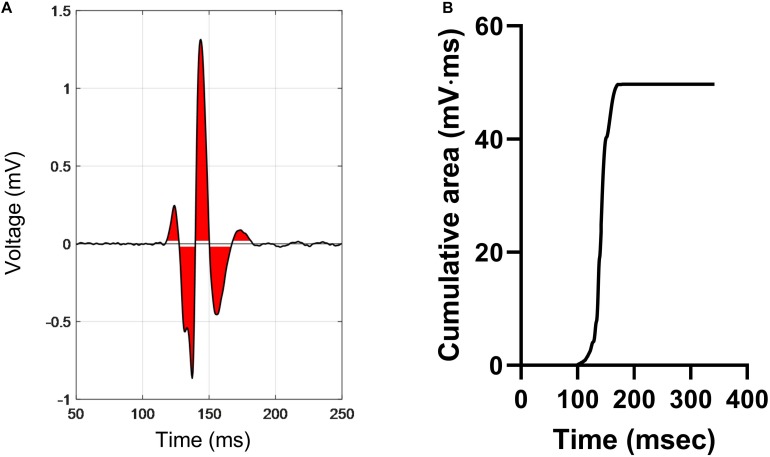
Calculation of electrogram area in a bipolar electrogram recording. **(A)** A representative bipolar electrogram recording. Signals above and below a noise threshold of ±0.05 mV are shaded in red. **(B)** Graph showing the cumulative sum of the shaded area, where the peak value is the total electrogram area. Normalized electrogram area is calculated by dividing the total area by the signal amplitude (maximum–minimum).

EA was derived by measuring the integrated area of the electrogram above and below a baseline noise threshold of ±0.05 mV over a fixed time window of 300 ms. A diagrammatic representation of the methodology is presented in Figure 2. The red area indicates the integrated area of the bipolar electrogram. The maximum value of the absolute cumulative area shown in Figure 2B equates to the total EA. norm_EA was calculated by dividing the total EA by the electrogram amplitude (maximum–minimum). Conduction delay was calculated from the difference in the activation time of the unipolar electrogram between each adjacent pair of electrodes, where activation time was taken as the time of minimum dV/dt of the unipolar signals.

### Statistical Analysis

Statistical comparisons were made by 1-way repeated measures ANOVA with Bonferroni *post hoc* tests. Statistical significance was taken as *p* < 0.05. Average data are presented as mean ± SEM with replicate values shown in gray.

## Results

### Computational Modeling

**FIGURE 3:**
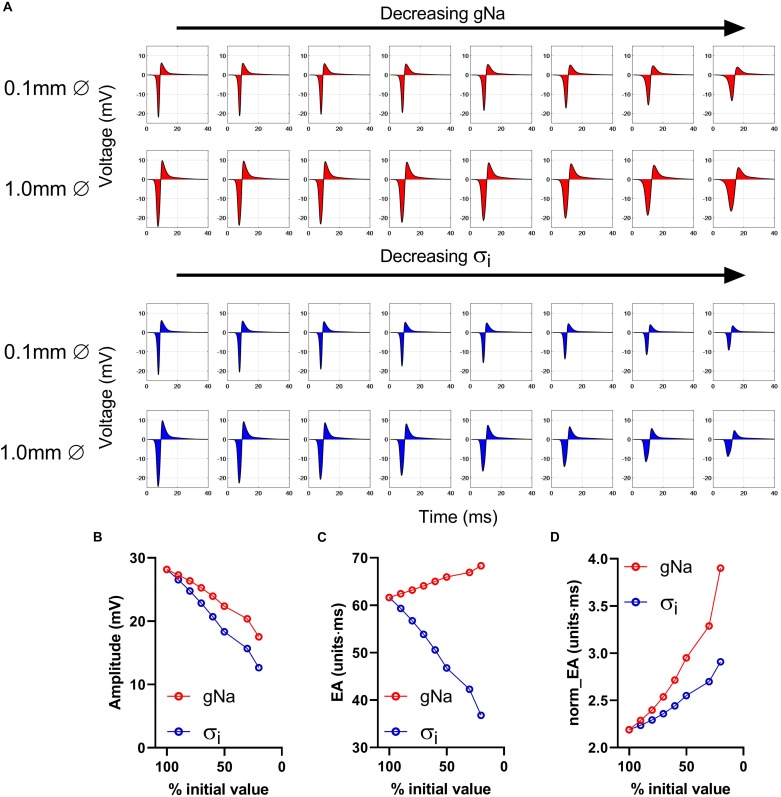
Impact of altering sodium channel conductance and intracellular conductivity on electrogram morphology in a mouse of a propagating mose ventricular action potential. **(A)** Simulated bipolar electrograms with decreasing sodium channel conductance (gNa) and intracellular conductivity (σi). Red shading shows the calculated electrogram area. Data are shown for electrode diameters of 0.1 and 1 mm. **(B–D)** Data showing changing signal amplitude **(B)**, absolute electrogram area (EA), **(C)**, and **(D)** normalized EA (norm_EA) as a % of gNa and σi (presented data for 0.1 mm electrode diameter).

**FIGURE 4:**
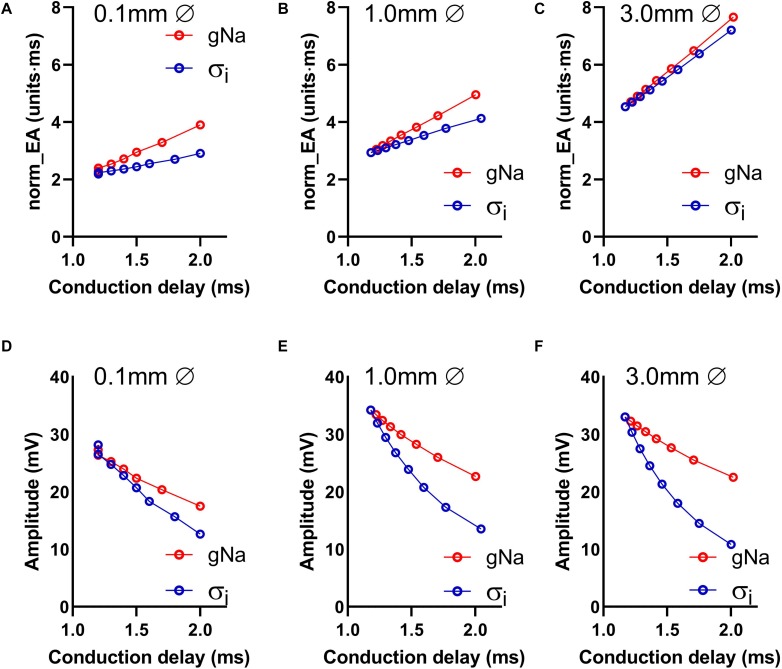
Influence of altered sodium channel conductance and intracellular conductivity on electrogram morphology and its relationship with local conduction delay. **(A–C)** Data showing the impact of altering electrode diameter on the relationship between normalized electrogram area (norm_EA) and local tissue conduction delay with altered sodium channel conductance (gNa)/intracellular conductivity (σ_*i*_) in a computational model of a propagating mouse ventricular action potential. Data are shown for three different electrode diameters. **(D–F)** The same data for bipolar electrogram amplitude.

**FIGURE 5:**
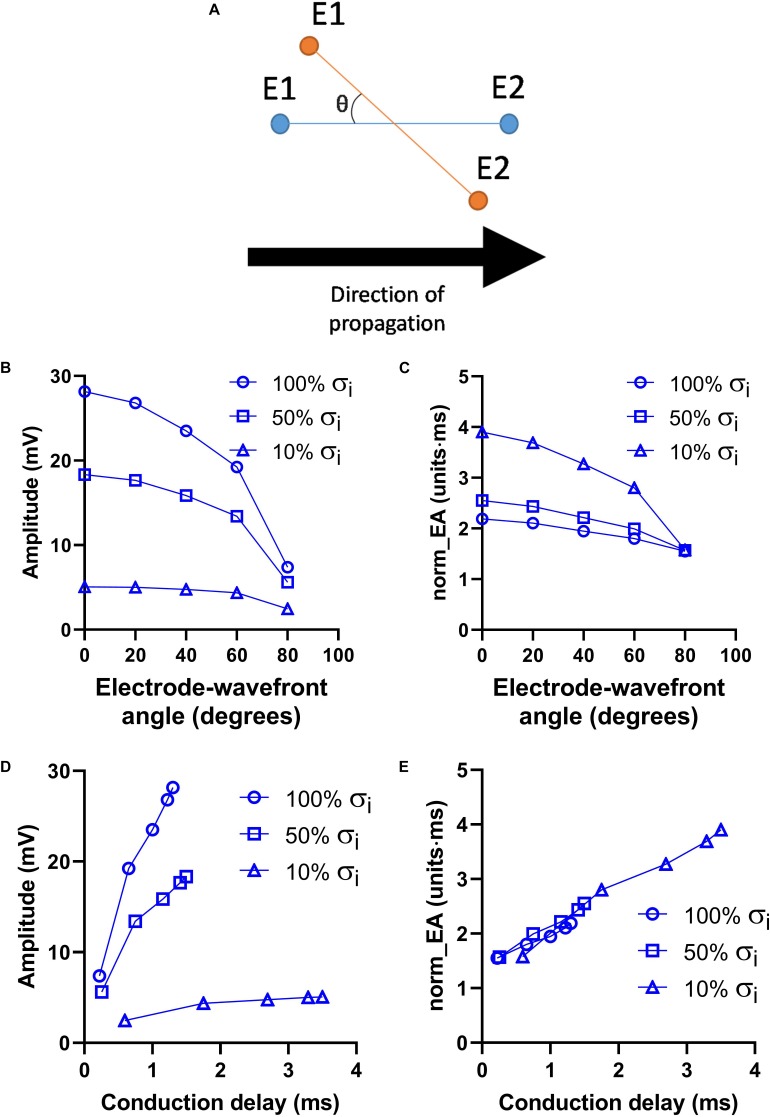
Influence of electrode orientation on bipolar electrogram morphology in a model of a propagating mouse action potential. Data showing the impact of altering the orientation of the recording electrodes relative to the direction of propagation of the activation wavefront (electrode-wavefront angle) on bipolar electrogram morphology. **(A)** Representative diagram showing change in electrode-wavefront angle. **(B,C)** Data showing the change in bipolar electrogram amplitude and normalized electrogram area (norm_EA) as a function of the electrode-wavefront angle. 0° represents the initial model conditions. Data are shown for varying levels of intracellular conductivity (σ_*i*_). **(D,E)** The same data plotted as a function of the local conduction delay between the recording electrodes.

The effects of conduction slowing via reduced gNa and σ_*i*_ were investigated using a computational model of a propagating mouse ventricular action potential (as described in the section “Materials and Methods”). Results for the simulations are presented in Figures 3–5. Traces in Figure 3A show simulated electrograms for varying levels of gNa and σ_*i*_ (as a % of initial model values). Decreasing either gNa or σ_*i*_ resulted in a reduction in bipolar electrogram amplitude and a broadening of electrogram morphology. The colored regions in Figure 3A indicate the electrogram area. Quantitative analysis of the change in signal amplitude and EA are shown in Figures 3B–D. A reduction in either model parameter led to a decrease in electrogram amplitude (Figure 3B), whereas altering gNa and σ_*i*_ had divergent effects on the absolute EA (Figure 3C)_._ The traces in Figure 3A show that whilst both parameters altered electrogram morphology, the effects on electrogram amplitude and duration were intervention-dependent. Altering gNa exerted a greater effect on electrogram duration vs amplitude and altering σ_*i*_ had a greater effect on electrogram amplitude vs duration. The change in absolute EA (Figure 3C) reflects the balance of these effects. Figure 3D shows absolute EA values normalized to electrogram amplitude, thus representing the amplitude-independent EA, norm_EA. norm_EA increased in response to reduced gNa and σ_*i*_, but to a greater degree in the former, consistent with more pronounced broadening of electrogram morphology for a given% change in gNa vs σ_*i*_.

Data presented in Figures 4A–C show the relationship between norm_EA and conduction delay between the two recording electrodes. Results are presented for electrode diameters of 0.1, 1.0, and 3.0 mm (electrode spacing was kept constant at 1 mm from edge-to-edge see Figure 1). Figure 4A presents data for the smallest recording electrode diameter (0.1 mm) and shows that the reduction of either gNa or σ_*i*_ caused an increase in the norm_EA as greater conduction delays are induced (slower conduction velocity). However, the absolute relationship between the variables differed for each intervention (Figure 4A). Corresponding norm_EA and conduction delay curves for simulations using larger electrode diameters are shown in Figures 4B,C. Notably, increasing the electrode diameter, which results in larger spatial signal averaging across the tissue, led to an increase in baseline norm_EA and the convergence of the gNa and σ_*i*_ curves (Figures 4B,C). Thus, at clinically relevant electrode sizes (1.0–3.0 mm), the norm_EA of a bipolar electrogram is a direct function of the conduction delay between the two recording electrodes. Notably, this is not the case for bipolar electrogram amplitude, as we found that increasing the electrode diameter led to greater divergence of the amplitude-conduction delay curves, as shown in Figures 4D–F.

Figure 5 presents data on the effects of altering the orientation of the recording electrodes relative to the direction wavefront propagation on electrogram morphology within the previously discussed model. The presented angles are relative to the initial orientation of the electrodes, as illustrated in Figure 5A. The effects of altering the electrode orientation from 0 to 90° on electrogram amplitude and norm_EA at varying levels of σ_*i*_ are shown in Figures 5A,B, respectively. As the electrode-wavefront angle was increased, both signal amplitude and norm_EA reduced (with no signal recorded when the electrodes were exactly perpendicular to the direction of wavefront propagation – data not shown). Similar results were observed for different levels of σ_*i*_, though the absolute magnitude and change in each variable differed for each simulation_._ The data shown in Figures 5D,E show the same data plotted against the local conduction delay between the electrode pair. Notably, whereas the relationship between signal amplitude and local conduction delay was non-linear and dependent on the level of σ_*i*_, the relationship between norm_EA and conduction delay was found to be linear. Thus, norm_EA was again found to be proportional to the local conduction delay between the electrode pair that make up the bipolar electrogram, irrespective of whether this delay was due to slowed conduction (i.e., via reduced σ_*i*_) or the orientation of the electrodes relative to the direction of the propagating activation wavefront.

**FIGURE 6:**
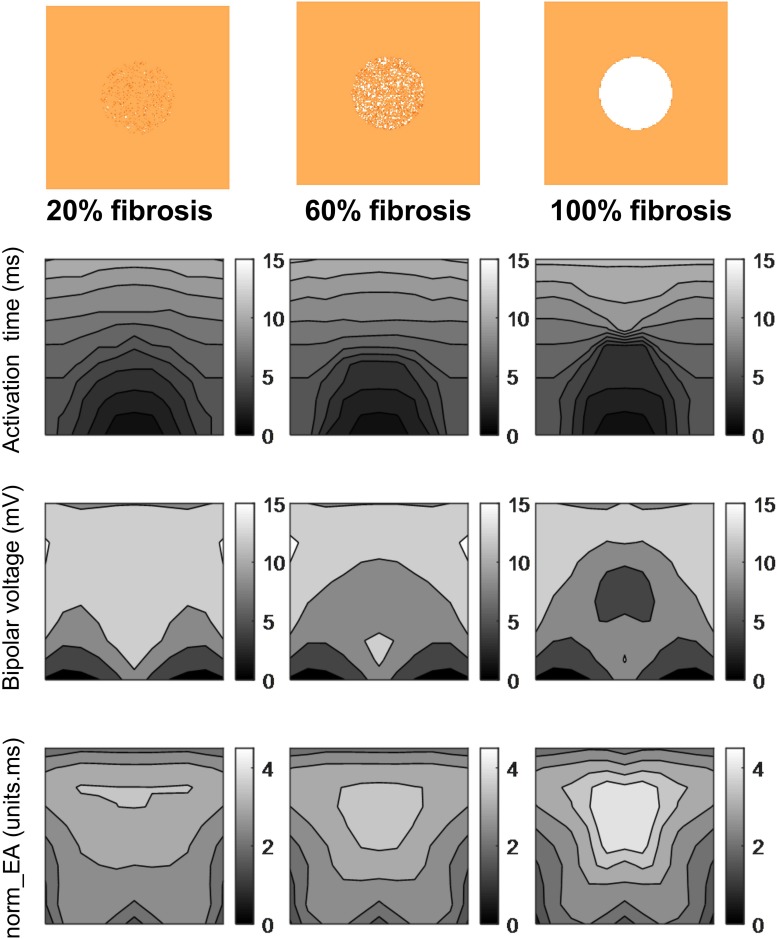
Influence of simulated fibrosis on bipolar electrogram morphology in a model of a propagating mouse action potential (maps). Maps showing the impact of simulated regional fibrosis on the pattern of electrical activation (as analyzed from a grid of unipolar electrograms), bipolar electrogram amplitude and normalized electrogram area (norm_EA).

**FIGURE 7:**
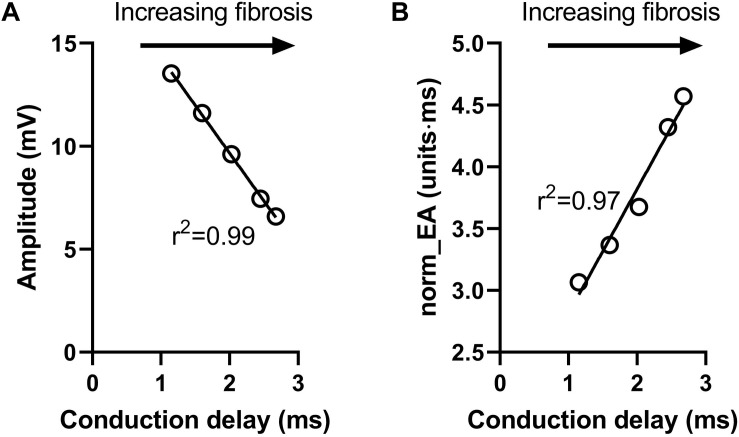
Influence of simulated fibrosis on bipolar electrogram morphology in a model of a propagating mouse action potential (analysis). Quantitative analysis of the relationship between conduction delay and electrogram morphology in conditions of varying levels of tissue fibrosis (electrogram recorded within fibrotic tissue). **(A)** Bipolar electrogram amplitude, **(B)** normalized electrogram area (norm_EA).

Figure 6 presents data on the impact of simulated regional fibrosis on the pattern of electrical activation and electrogram morphology. Activation (derived from a 10 × 10 grid of unipolar signals), and calculated bipolar voltage and norm_EA maps are shown for varying degrees of fibrosis (20–100%) within a circular region of tissue. Increasing levels of fibrosis were associated with slowing of conduction (as evidenced by the crowding of the isochronal lines), a reduction in bipolar electrogram amplitude and a decrease in norm_EA. Notably, regional electrograms were observed even at 100% fibrosis, as finite elements labeled as fibrotic are only removed from the intracellular grid. Quantitative analysis of electrograms recorded within the fibrotic core region showed a linear reduction in amplitude and increase in norm_EA as a function of increasing conduction delay, as presented in Figure 7. Visual inspection shows greater spatial association between fibrotic regions and increased norm_EA than amplitude, although these associations were not quantified in this study.

### Experimental Studies

#### Studies in Isolated Mouse Hearts

**FIGURE 8:**
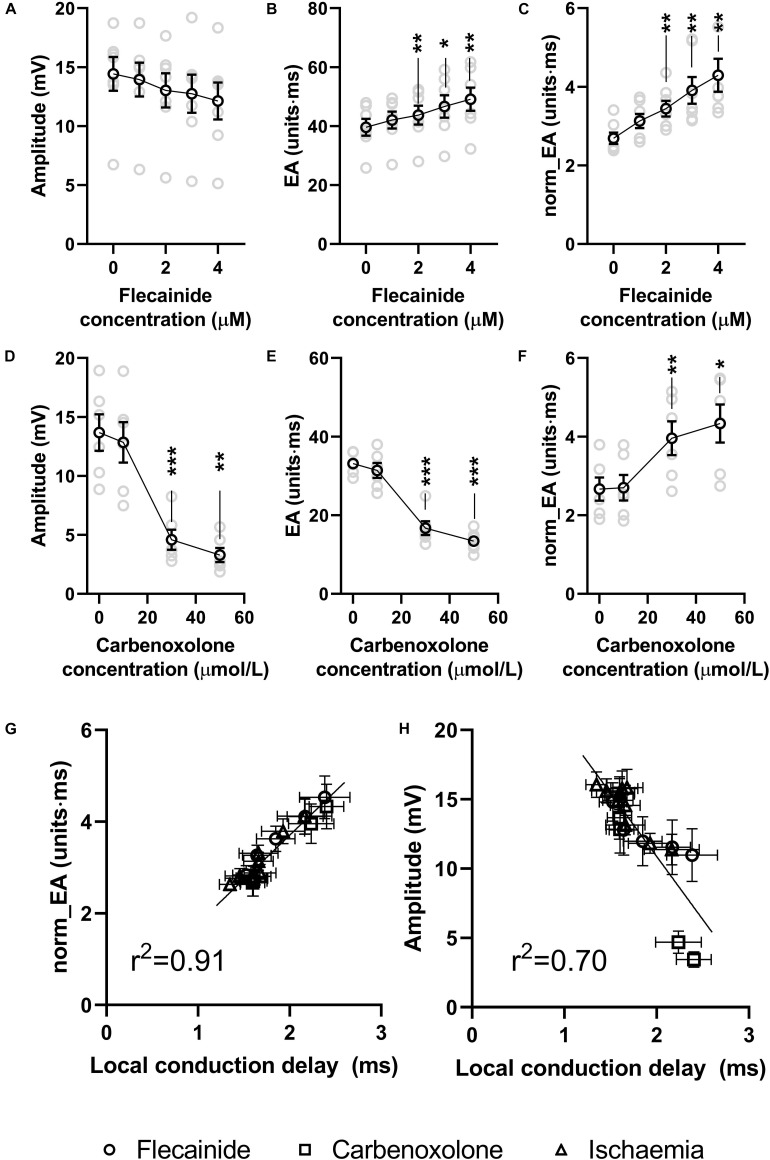
Effects of sodium channel blockade and gap junction inhibition on electrogram morphology in isolated mouse hearts. **(A–C)** Data showing the effects of increasing concentrations of flecainide on bipolar electrogram amplitude, absolute electrogram area (EA) and normalized EA (norm_EA). **(D–F)** The same panels but for increasing concentrations of carbenoxolone. **(G,H)** Correlation between norm_EA and local activation delay (as assessed from the difference in activation time between adjacent electrodes/unipolar electrograms). Difference from 0 μmol/L; *p < 0.05, **p < 0.01, ***p < 0.001. Data are mean ± SEM. Actual replicates are shown in gray. *n* = 6,7 hearts per group.

Data presented in Figure 8 show the effects of the sodium channel blocker flecainide and gap junction inhibitor carbenoxolone on the morphology of bipolar electrograms recorded from the endocardial surface of the mouse left ventricle. This replicates experimentally the effects of reduced gNa and σ_*i*_ in the computational model. Data showing concentration-dependent changes in bipolar amplitude, absolute EA and norm_EA are presented in panels a–c for flecainide and panels d–e for carbenoxolone. At 30 μmol/L, carbenoxolone caused a substantive decrease in electrogram amplitude. A similar, but smaller, effect was observed with flecainide. One outlier result prevented this effect being statistically significant. Consistent with the computational simulations, the concentration-dependent change in absolute EA was different for each treatment, increasing in response to flecainide and decreasing with carbenoxolone. Meanwhile, norm_EA increased in a concentration-dependent manner for both treatments.

**FIGURE 9:**
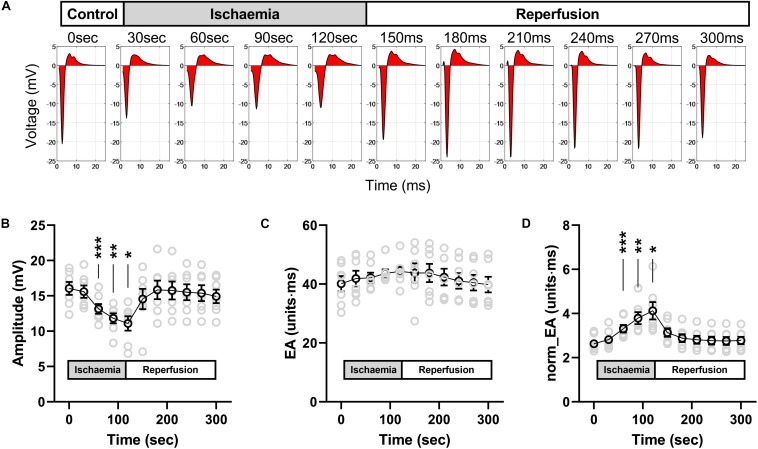
Effects of low-flow ischemia on electrogram morphology in isolated mouse hearts. Changes in electrogram morphology during low-flow global ischemia in perfused mouse hearts. **(A)** Representative bipolar electrogram recordings showing the change in electrogram morphology during a 120-s period of low-flow ischemia and subsequent tissue reperfusion in a single mouse heart. The red shading indicates the calculated electrogram area. **(B–D)** Mean data from seven mouse hearts showing the changes in bipolar electrogram amplitude **(B)**, absolute electrogram area (EA) **(C)**, and amplitude-normalized EA (norm_EA) **(D)** in response to ischemia-reperfusion. Different from 0; *p < 0.05, **p < 0.01, ***p < 0.001. Data are mean ± SEM. Actual replicates are shown in gray. *n* = 7 hearts.

Figure 9 presents data on the impact of low-flow global ischemia and reperfusion on electrogram morphology in the mouse heart; a pathophysiological cause of conduction slowing. Figure 9A shows the changes in electrogram morphology associated with a 2-min period of low-flow ischemia at 25% of the initial flow rate. During low-flow perfusion, a reduction in amplitude and broadening of the bipolar electrogram was observed, which rapidly recovered to initial values on tissue reperfusion. Summary data for changes in signal amplitude, absolute EA and norm_EA are shown in Figures 9B–D. The results are consistent with the effects of reduced sodium channel availability. Ischemia was associated with a reduction in signal amplitude, no change in absolute EA and an increase in norm_EA. All values normalised on tissue reperfusion.

**FIGURE 10:**
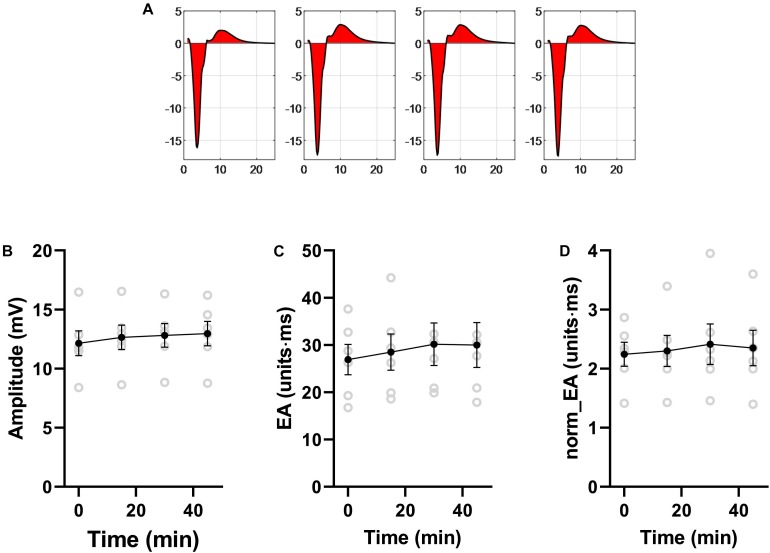
Stability of bipolar electrogram metrics in isolated mouse hearts. Data showing the stability of measures of electrogram morphology in isolated mouse hearts. EA = electrogram area. norm_EA = amplitude-normalized electrogram area. *n* = 6 hearts.

Data presented in Figure 10 shows that norm_EA and signal amplitude are stable in experimental recordings made without the study interventions, indicating that the changes observed for flecainide, carbenoxolone and ischemia were due to their direct biological action.

Figure 8G shows the relationship between norm_EA and local conduction delay; as measured from the mean difference in activation time between adjacent unipolar electrogram recordings. A strong linear correlation was observed (*r*^2^ = 0.92, *p* < 0.0001), with data from low-flow ischemia, flecainide and carbenoxolone protocols falling on the same linear relationship. In contrast, the relation between bipolar electrogram amplitude and local conduction delay diverges from a linear relationship (panel h, *r*^2^ = 0.70, *p* < 0.0001).

#### Studies in Isolated Guinea Pig Hearts

**FIGURE 11:**
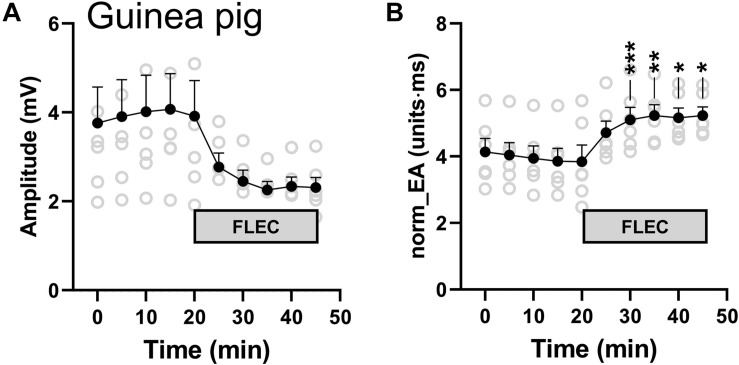
Influence of flecainide on electrogram morphology in perfused guinea pig hearts. **(A)** Mean data from 6 experiments showing the effects of switching to buffer containing 4 μmol/L flecainide on bipolar electrogram amplitude and amplitude-normalized electrogram area (norm_EA). Different from time 0; *p < 0.05, **p < 0.01, ***p < 0.001. Data are mean ± SEM. Actual replicates are shown in gray. *n* = 6 hearts.

We next investigated the impact of sodium channel block on bipolar norm_EA in isolated perfused guinea pig hearts, which have similar action potential morphology and ion channel expression as that of the human heart. Guinea pig hearts, beating at their intrinsic rate, were perfused with a standard crystalloid buffer, before switching to a buffer solution containing 4 μmol/L of flecainide. A marked reduction in amplitude and increase in norm_EA was observed, which is shown in in Figures 11A,B, and is consistent with those observed in perfused mouse hearts (Figure 8).

## Discussion

Using computational modeling and experimental studies performed in isolated mammalian hearts, the present study presents data in support of a novel metric of local conduction delay in the heart – that of the norm_EA of a bipolar elecrogram. In mouse hearts, we found that the change in norm_EA was directly proportional to the change in local conduction delay between closely spaced (bipolar) recording electrodes and that this relationship was independent of the mechanism by which a change in conductuin delay was achieved (i.e., sodium channel block, gap junction inhibition and ischemia). Meanwhile, bipolar electrogram amplitude, a commonly used metric in substrate mapping procedures, was found to be differentially impacted by the effects of sodium channel block and gap junction inhibition. Whilst altering electrode orientation had a marked impact on electrogram morphology in our simulation studies, our results indicate that the change in norm_EA remains proportional to the degree of conduction delay between the recording electrodes, which is of course influenced by tissue conduction velocity, but also the spacing between the electrodes and their position relative to the activation wavefront. This finding remained true for a range of model parameters, which was notably not the case for bipolar electrogram amplitude. On the basis of these results, we conclude that the norm_EA of a bipolar electrogram is a quantitative index of temporal difference in activation time of the tissue near to the recording electrodes, at least in the mouse heart. We also recorded qualitatively similar changes in electrogram morphology in guinea pig hearts perfused with flecainide, as those observed in the mouse heart, suggesting our findings may be more broadly applicable to other species.

Theoretically, an increase in conduction delay in the heart will result in a broadening of the QRS-morphology of an electrogram recorded from two closely spaced poles (i.e., a bipolar electrogram). Local conduction delay could therefore be quantified by measuring the duration of the corresponding electrograms. However, whilst electrogram duration is relatively easy to measure for electrograms recorded from healthy tissue, it becomes more difficult to define accurately for the low-amplitude, multiphasic signals often recorded in and around fibrotic and scarred tissue. A major limitation is that electrogram duration depends on the correct placement of only two points relative to the activation front, and, therefore it is particularly sensitive to errors caused by signal artifacts and noise, resulting in incorrect labeling of the start and end of the electrogram complexes. Such ambiguity is avoided (or at least limited) in the norm_EA metric, where integration of the total electrogram area is less sensitive to both signal artifacts and noise. Notably, the norm_EA metric is also easy to calculate and computationally inexpensive, and so could be easily integrated into existing electroanatomical mapping platforms/software. However, it is recognized that the total area of the electrogram is not simply a function of amplitude and duration, but of the overall morphology of the electrogram, including the number, shape and relative size of the peaks and troughs within the signal. Thus, norm_EA is not a direct surrogate for electrogram duration, but a complex measure that is influenced by multiple factors. In the present study, we have generated evidence showing that the norm_EA is directly proportional to the conduction delay between the recording electrodes of a bipolar electrogram. Notably, similar relationships have not been established for other substrate mapping metrics.

In simulation studies, we found that delays in activation caused by fibrosis [modeled as different densities (20–100%) of non-conducting tissue] led to a linear decrease and increase in signal amplitude and norm_EA, respectively, when considering electrograms in the centre of the fibrotic region. This would imply that both bipolar electrogram amplitude and norm_EA are accurate measures of local conduction delays due to fibrosis and the resulting tortuous pattern of activation through the remaining tissue. However, norm_EA appears to better correlate spatially with regional fibrosis than amplitude (Figure 6), although we did not quantify this relationship. Determining this spatial correlation would require more complex computational models, which account for realist infarct scar morphology and wall thickness, defining voltage and norm_EA thresholds, and validating these against clinical data of chronic infarct patients (Mukherjee et al., 2019). Such detailed investigation is out of the scope of this proof-of-concept study.

Whilst the present study does not address the efficacy of the norm_EA metric in substrate mapping procedures, clinical testing is a clear goal of future research. Instead, the present work provides good evidence in support of the norm_EA metric in the assessment of local conduction delay in the heart, which feasibly has utility for the mapping of pro-arrhythmogenic substrate in non-inducible and haemodynamically unstable arrhythmia, such as scar-related ventricular tachycardia.

## Conclusion

Using a combined computation and experimental approach, this study provides evidence that norm_EA of a bipolar electrogram is a quantitative index of local conduction delay in the tissue close to the recording electrodes. This novel metric may have utility in electrophysiological substrate mapping procedures, but further validation in a model of chronic myocardial infarction is required.

## Financial Disclosures

PK receives research support for basic, translational, and clinical research projects from European Union, British Heart Foundation, Leducq Foundation, Medical Research Council (United Kingdom), and German Center for Cardiovascular Research, from several drug and device companies active in atrial fibrillation, and has received honoraria from several such companies in the past. PK is listed as inventor on two patents held by University of Birmingham (Atrial Fibrillation Therapy WO 2015140571, Markers for Atrial Fibrillation WO 2016012783).

## Data Availability Statement

The datasets generated for this study are available on request to the corresponding author.

## Ethics Statement

The animal study was reviewed and approved by Birmingham University and King’s College London Animal Welfare and Ethics Review Committees.

## Author Contributions

JW and DP developed the concept and designed the experiments. GA, VM, CO’S, DP, and JW conducted the experimental studies and analyzed the results. CM and SN conducted the computational simulations. PK, RC, and TD provided expert advice and direction. All authors reviewed and contributed to the final manuscript.

## Conflict of Interest

PK receives research support for basic, translational, and clinical research projects from European Union, British Heart Foundation, Leducq Foundation, Medical Research Council (United Kingdom), and German Center for Cardiovascular Research, from several drug and device companies active in atrial fibrillation, and has received honoraria from several such companies in the past. PK is listed as inventor on two patents held by University of Birmingham (Atrial Fibrillation Therapy WO 2015140571, Markers for Atrial Fibrillation WO 2016012783). The remaining authors declare that the research was conducted in the absence of any commercial or financial relationships that could be construed as a potential conflict of interest. The handling Editor declared a past co-authorship with one of the authors RC.
